# Association between prognostic nutritional index and prognosis in non-small cell lung cancer patients receiving PD-1/PD-L1 inhibitors: a meta-analysis

**DOI:** 10.3389/fnut.2026.1859449

**Published:** 2026-07-23

**Authors:** Ping Li, Zaoke Wu, Han Chen, Hanchao Zhang, Zhumei Luo

**Affiliations:** 1 Department of Oncology, The Third People’s Hospital of Chengdu, Affiliated Hospital of Southwest Jiaotong University, Chengdu, Sichuan, China; 2 Department of Urology, Sichuan Provincial Construction Hospital, Chengdu, Sichuan, China; 3 Department of Urology, The Affiliated Hospital and Clinical Medical College of Chengdu University, Chengdu, Sichuan, China

**Keywords:** meta-analysis, non-small cell lung cancer, PD-1, PD-L1, prognostic nutritional index

## Abstract

**Objective:**

This study aimed to comprehensively evaluate the predictive value of prognostic nutritional index (PNI) for long-term survival in non-small cell lung cancer (NSCLC) patients treated with programmed death receptor-1 and its ligand (PD-1/PD-L1) inhibitors through systematic review and meta-analysis.

**Methods:**

A systematic search was conducted in PubMed, Embase, Web of Science, and the Cochrane Library databases up to January 2026. Observational studies assessing the association between PNI and survival outcomes in NSCLC patients treated with PD-1/PD-L1 inhibitors were included. Two investigators independently performed literature screening, data extraction, and methodological quality assessment (using the Newcastle-Ottawa Scale). Heterogeneity was assessed using the pooled hazard ratio (HR) and its 95% confidence interval (CI) in a random-effects model, and subgroup analyses, sensitivity analyses, and publication bias assessments were performed.

**Results:**

A total of 14 studies involving 2,106 patients were included. Meta-analysis results showed that a higher PNI was associated with significantly improved overall survival (OS) (HR = 0.46, 95% CI: 0.34–0.64, *p* < 0.00001) and progression-free survival (PFS) (HR = 0.63, 95% CI: 0.52–0.76, p < 0.00001). Subgroup analysis indicated that the prognostic value of PNI remained consistent regardless of study region (China, Turkey, Japan, UK) or PNI cutoff value (>45 or ≤45). OS analysis showed high heterogeneity (I^2^ = 89%), but sensitivity analysis results were robust. Publication bias assessment suggested publication bias in OS, but after correction using pruning and complementation, the significant association between PNI and OS remained unchanged (corrected HR: 0.47, 95% CI: 0.34–0.64).

**Conclusion:**

Current evidence suggests an association between higher baseline PNI and improved survival outcomes in NSCLC patients receiving PD-1/PD-L1 inhibitor therapy. The PNI may serve as a candidate biomarker for risk stratification, but its clinical utility requires validation in prospective studies.

## Introduction

Non-small cell lung cancer (NSCLC), the most prevalent pathological type of lung cancer, accounts for approximately 85% of all lung cancer cases (1, 2). Its high incidence and mortality rates make it a major global public health challenge (1, 3, 4). Despite continuous advancements in traditional treatments such as surgery, radiotherapy, and chemotherapy, the prognosis for patients with advanced NSCLC has long remained unsatisfactory (3, 5, 6). In recent years, tumor immunotherapy, particularly immune checkpoint inhibitors (ICIs) targeting programmed death receptor-1 (PD-1) and its ligand (PD-L1), has fundamentally changed the treatment landscape for advanced NSCLC (7, 8). However, immunotherapy is not effective for everyone; its objective response rate still has room for improvement, and it is accompanied by unique immune-related adverse events (9, 10). Therefore, accurately identifying patients most likely to benefit from ICIs is one of the core issues that urgently need to be addressed in the field of tumor immunotherapy (11).

Systemic inflammatory response and nutritional status in patients are considered to play a crucial regulatory role in anti-tumor immune responses, and therefore have received widespread attention as readily available and inexpensive potential predictive indicators (12, 13). Chronic inflammation not only promotes tumor development and progression but may also shape an immunosuppressive tumor microenvironment, weakening the effectiveness of immunotherapy (14–16). Malnutrition and cancer cachexia are closely associated with decreased immune function, poor treatment tolerance, and poor prognosis (17, 18). The prognostic nutritional index (PNI) is a composite indicator reflecting the body’s nutritional status and inflammation level, calculated as: PNI = Serum albumin (g/L) + 5 × Total peripheral blood lymphocytes (×10^9^/L) (19, 20). A decreased PNI value may indicate that the patient is in an unfavorable state of malnutrition, systemic inflammation, and lymphopenia (21). Theoretically, this state would impair the host’s immune capacity against tumors, potentially weakening the efficacy of ICIs and leading to poor survival outcomes (17, 22).

While PNI has been demonstrated in various solid tumors such as gastric and colorectal cancer, its predictive role in NSCLC within the context of immunotherapy remains unclear and lacks consensus among current evidence (23–26). In recent years, numerous studies both domestically and internationally have explored the relationship between PNI and survival outcomes in NSCLC patients treated with PD-1/PD-L1 inhibitors (21, 27–29). Most of these studies are retrospective, covering multiple regions including Turkey, China, Japan, and the UK. Most studies have reported a significant association between lower PNI and shorter overall survival (OS) and/or progression-free survival (PFS), supporting its role as a poor prognostic factor (21, 29, 30). However, differences in population characteristics, PNI measurement time points, and statistical analysis methods among different studies have led to varying effect estimates and potentially conflicting conclusions. Although previous meta-analyses have explored the association between PNI and survival outcomes in lung cancer patients treated with ICIs (31), this study’s search ended in August 2023, and it combined two lung cancer types with significant differences in pathogenesis, treatment modalities, and prognostic characteristics, which may have somewhat obscured the precise predictive efficacy of PNI in specific populations (31). Furthermore, the study did not conduct in-depth subgroup analysis on key factors that could lead to heterogeneity, such as the type of ICI, differences in the study region, and the different PNI threshold values used (31).

Therefore, this study aims to provide a more focused, in-depth, and up-to-date synthesis of evidence. We specifically targeted the NSCLC patient population, updating the literature search to January 2026 to include the latest research evidence. In addition to validating the overall predictive value of PNI for OS and PFS, we innovatively conducted subgroup analyses based on the study’s geographical location and PNI threshold to explore the potential impact of these factors on the prognostic efficacy of PNI. Furthermore, we employed a pruning and complement method to correct for identified publication bias, enhancing the robustness of our conclusions. Through these design elements, this study aims to provide a more timely, comprehensive, and methodologically rigorous evidence map for the application of PNI in the specific clinical scenario of NSCLC immunotherapy.

## Methods

### Literature search

This meta-analysis was conducted in accordance with the PRISMA 2020 statement (32) and was registered in PROSPERO (CRD420261372560). To identify relevant studies assessing the association between PNI and prognosis in NSCLC patients receiving PD-1/PD-L1 inhibitors, a systematic literature search was performed in PubMed, Embase, Web of Science, and the Cochrane Library up to January 2026. The search query in PubMed is as follows: (((“Carcinoma, Non-Small-Cell Lung”[Mesh]) OR (((((((Non-Small-Cell Lung Carcinomas) OR (Non-Small Cell Lung Cancer)) OR (Non-Small-Cell Lung Carcinoma)) OR (Non Small Cell Lung Carcinoma)) OR (Nonsmall Cell Lung Cancer)) OR (Non-Small Cell Lung Carcinoma)) OR (NSCLC))) AND ((“Immune Checkpoint Inhibitors”[Mesh]) OR (((((((((((Programmed Cell Death Protein 1 Inhibitor) OR (PD-1 Inhibitor)) OR (PD 1 Inhibitor)) OR (Programmed Death-Ligand 1 Inhibitors)) OR (Programmed Death Ligand 1 Inhibitors)) OR (PD-L1 Inhibitor)) OR (PD L1 Inhibitor)) OR (PD-1)) OR (PD-L1)) OR (PD 1)) OR (PD L1)))) AND ((prognostic nutritional index[Title/Abstract]) OR (PNI)). Detailed search strategies of all databases are presented in Supplementary Table 1. To ensure completeness, the bibliographies of all included articles were manually screened. The process of article selection and evaluation was conducted independently by two investigators, and any discrepancies were resolved through discussion.

### Inclusion and exclusion criteria

*Inclusion criteria*:

P: Patients diagnosed with NSCLC and receiving PD-1/PD-L1 inhibitor therapy.
E: High PNI.
C: Low PNI.
O: Any survival outcome, including overall survival (OS), and progression-free survival (PFS), etc.
S: Study design was cohort or case–control.

*Exclusion criteria*: studies that did not meet the following criteria were excluded: protocols, unpublished data, and non-original publications including letters, abstracts, commentaries, corrections, and replies. Single-arm trials, review articles, and studies with insufficient data-particularly those in which hazard ratios (HRs) and 95% confidence intervals (CIs) for survival outcomes could not be extracted or calculated-were also omitted. This systematic review only included studies published in English; studies published in other languages were not searched or screened.

### Data abstraction

Data extraction was performed independently by two investigators, with any disagreements adjudicated by a senior researcher. The extracted data encompassed the first author’s name, publication year, geographic region, study design, population characteristics, sample size, patient demographics, cut-off values, as well as HRs (after adjustment using a multivariate Cox proportional hazards model) and 95% CIs for OS and PFS. Corresponding authors were contacted to obtain any missing or unclear data.

### Quality evaluation

The quality of the included observational studies (cohort and case–control designs) was appraised with the Newcastle-Ottawa Scale (NOS) (2). A predefined threshold score of 7 to 9 was used to classify studies as high quality (33). This appraisal followed a dual-reviewer process conducted independently, with any divergences in scoring resolved by consensus discussion.

### Statistical analysis

The analysis was performed with Review Manager software (v5.4.1). Survival data were synthesized using pooled HRs with 95% CIs, based on a random-effects model. Study heterogeneity was quantified with Cochrane’s Q test and the I^2^ inconsistency index (34, 35) a threshold of *p* < 0.10 for the Q test and I^2^ > 50% defined high heterogeneity. Sensitivity analysis was employed for outcomes derived from more than two studies to evaluate the robustness of the results. Subgroup analyses, stratified by study region and cutoff value, were conducted to investigate sources of significant heterogeneity. Potential publication bias was examined using Egger’s regression test (36) in Stata 15.1 for outcomes encompassing ten or more studies; a *p* value < 0.05 was considered statistically significant. For outcomes showing evidence of publication bias, the trim-and-fill method was used to adjust the estimates.

## Results

### Literature retrieval and study characteristics

**Figure 1:**
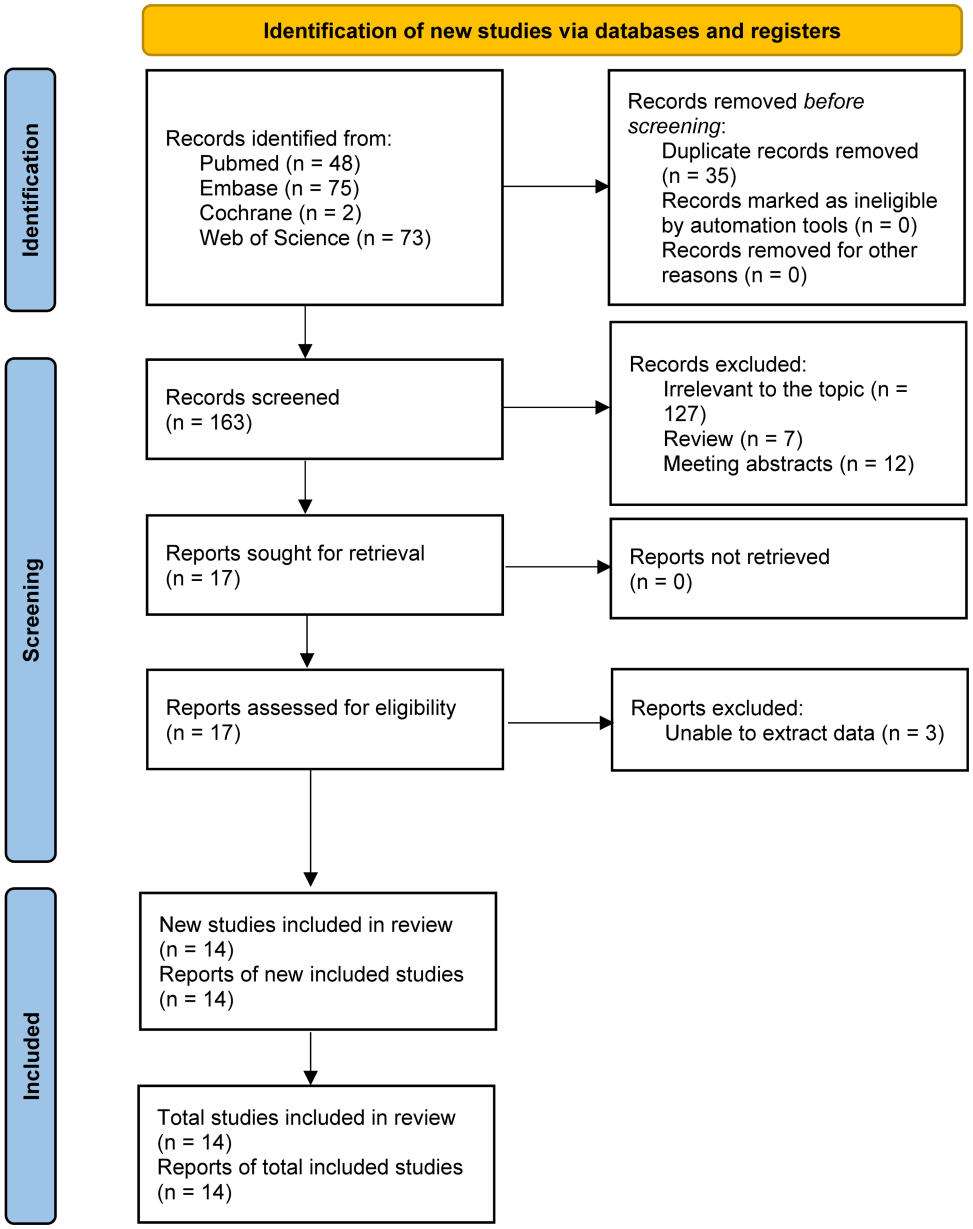
Flowchart of the systematic search and selection process.

The process of literature identification and selection is illustrated in Figure 1. A comprehensive search across PubMed (*n* = 48), Embase (*n* = 75), Web of Science (*n* = 73), and the Cochrane Library (*n* = 2) initially yielded a total of 198 records. After duplicate entries were removed, 163 unique titles and abstracts were screened for relevance. In the final analysis, 14 studies encompassing a total of 2,106 patients were deemed eligible and included in the meta-analysis (21, 27–30, 37–45). Table 1 summarizes the key features and methodological quality of each eligible study. The included literature was published between 2020 and 2026.

**Table 1:** Characteristics and quality assessment of included studies.

Author	Region	Study design	Population	No. of patients	Gender		Mean/median age	PNI cut-off	NOS
					Male	Female			
Alan et al. (29) (2025)	Turkey	Retrospective	NSCLC patients treated with second-line nivolumab	196	167	29	65	45.2	7
Beypınar et al. (21) (2026)	Turkey	Retrospective	Non-small cell lung cancer patients receiving nivolumab immunotherapy	199	174	25	63.4	43.5	7
Fang et al. (30) (2023)	China	Retrospective	Advanced NSCLC patients receiving PD-1 inhibitor therapy	223	189	34	NA	NA	8
Hashimoto et al. (28) (2025)	Japan	Retrospective	Patients with advanced NSCLC who received nivolumab plus ipilimumab	110	89	21	NA	42.6	8
Jiang et al. (37) (2024)	China	Retrospective	Advanced non-small cell lung cancer patients receiving PD-1 inhibitors	184	142	42	NA	46.67	8
Liu et al. (38) (2021)	China	Retrospective	Advanced Non-small Cell Lung Cancer patients treated with PD-1 inhibitors	123	98	25	NA	46.05	8
Matsubara et al. (39) (2020)	Japan	Retrospective	Non-small cell lung cancer patients treated with atezolizumab	24	17	7	64.5	40	8
Nishihara-Kato et al. (40) (2024)	Japan	Retrospective	Non-Small Cell Lung Cancer Patients Receiving Pembrolizumab Combination Therapy with Carboplatin and Paclitaxel/ Nab-Paclitaxel	144	121	23	69	45	8
Peng et al. (41) (2020)	China	Retrospective	Advanced non‑small cell lung cancer treated with PD‑1 inhibitors	102	87	15	NA	45	7
Stares et al. (42) (2022)	UK	Retrospective	Patients treated with first-line pembrolizumab for advanced NSCLC with programmed death-ligand 1 expression 50%	219	109	110	NA	45	7
Tang et al. (43) (2024)	China	Retrospective	Advanced NSCLC patients treated with anti-PD-1 drugs	148	131	17	NA	45	8
Ulas et al. (44) (2024)	Turkey	Retrospective	Advanced Non-Small-Cell Lung Cancer Treated with Nivolumab	104	94	10	62	40.08	7
Yamaguchi et al. (45) (2024)	Japan	Retrospective	Patients with advanced NSCLC who received nivolumab plus ipilimumab	101	83	18	NA	NA	7
Zeynelgil et al. (27) (2025)	Turkey	Retrospective	Metastatic Non-Small Cell Lung Cancer Patients Receiving Nivolumab	229	193	36	NA	45.1	7

### Prognostic value of PNI for OS

**Figure 2:**
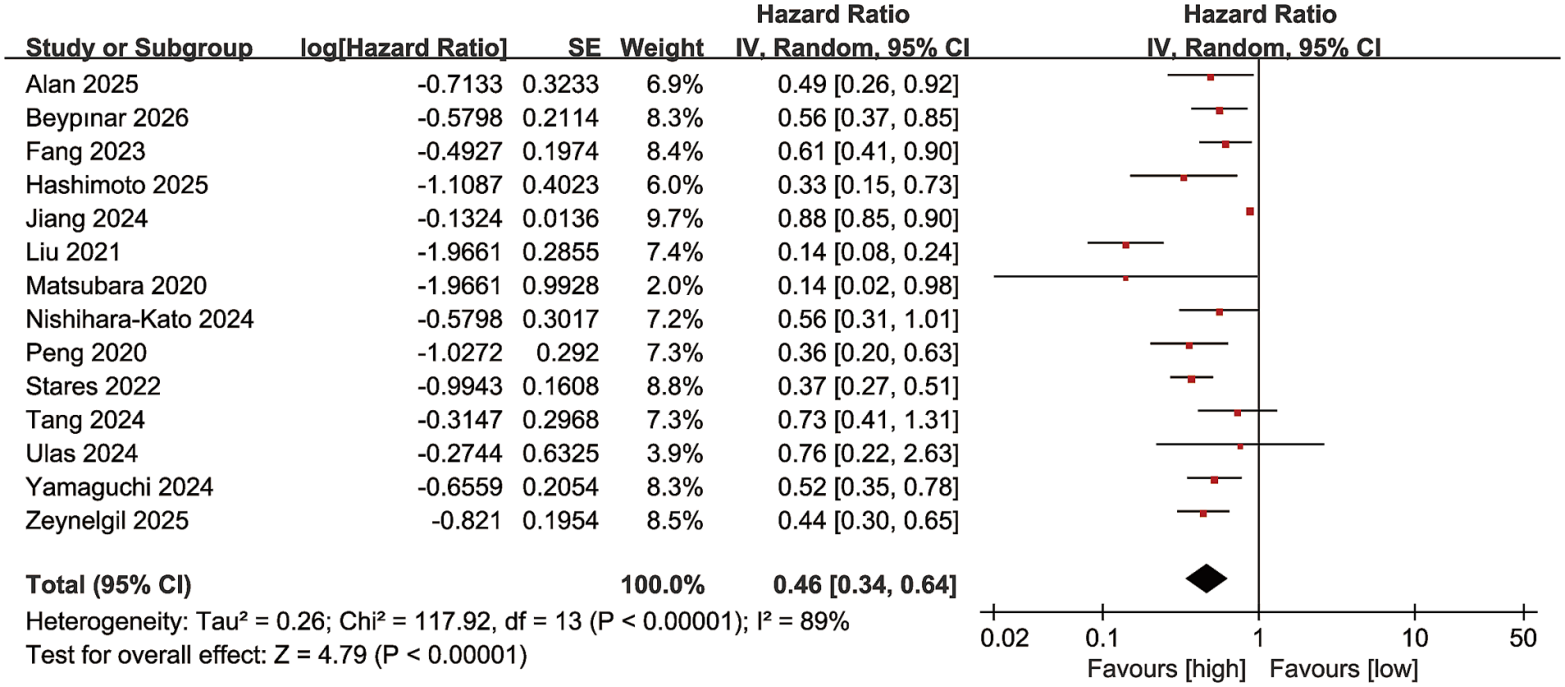
Forest plots of the prognostic value of PNI for OS.

**Figure 3:**
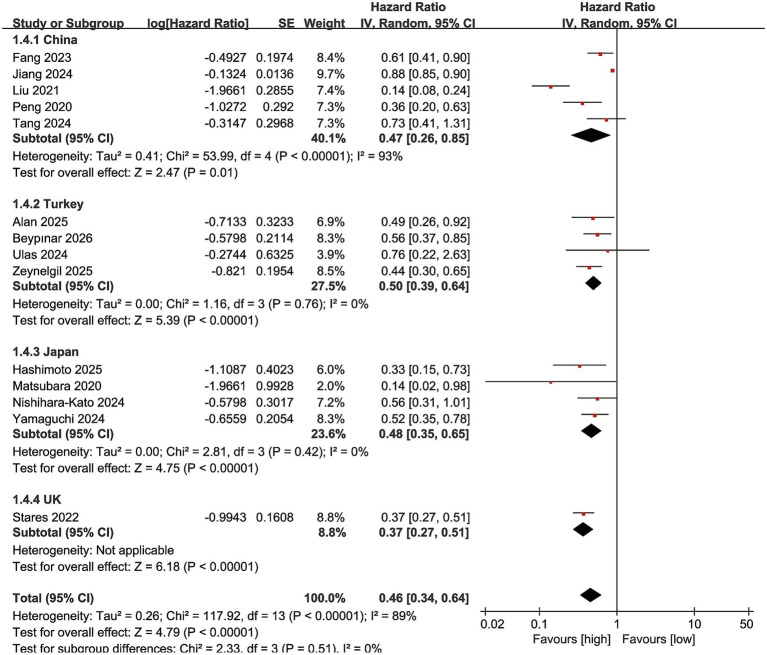
Region-based subgroup analysis of the prognostic value of PNI for OS.

**Figure 4:**
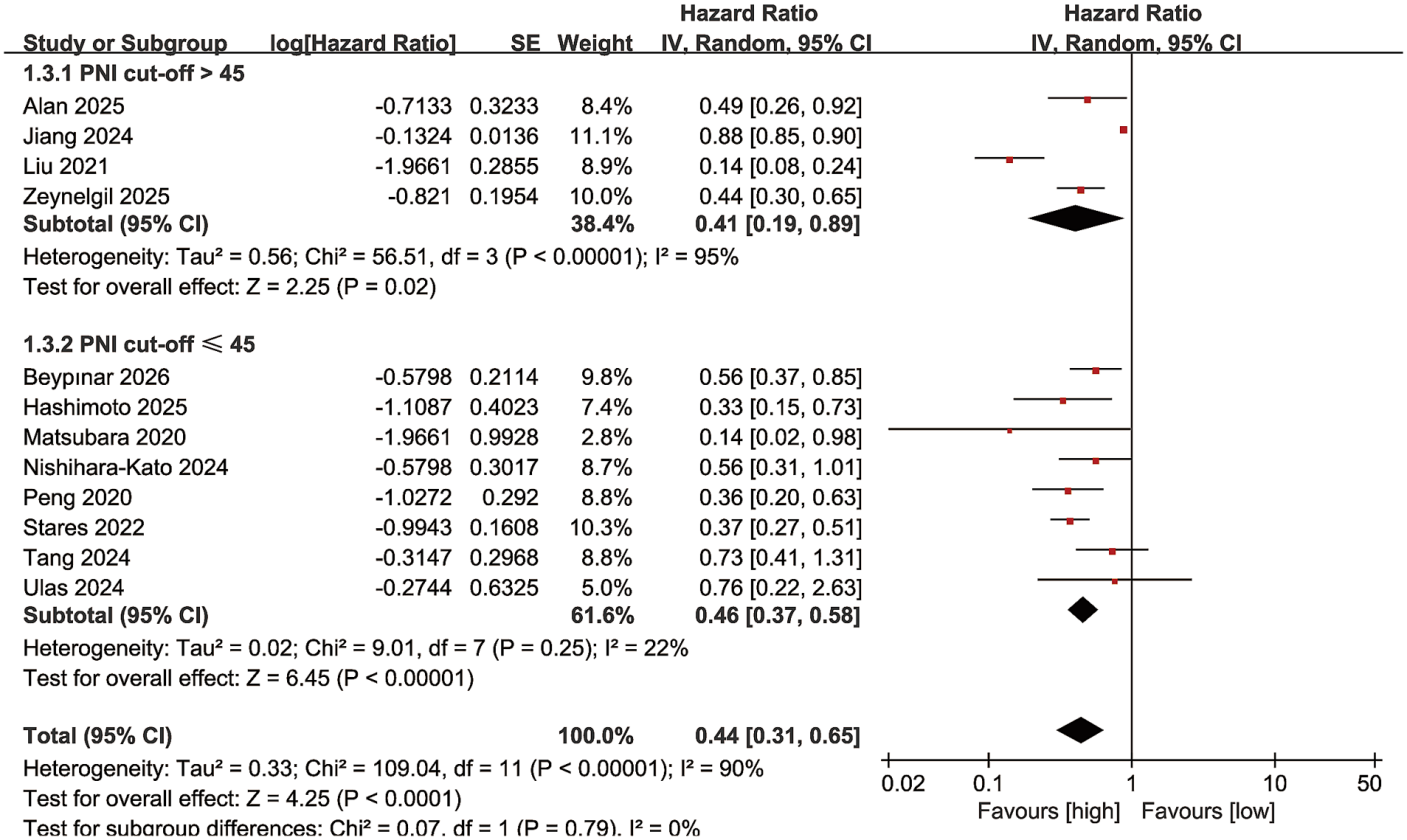
PNI cutoff-based subgroup analysis of the prognostic value of PNI for OS.

OS results were synthesized from 14 studies. The meta-analysis indicated that a higher PNI was significantly associated with longer OS (HR: 0.46; 95% CI: 0.34, 0.64; *p* <0.00001), with a substantial heterogeneity (*I*^2^ = 89%, *p* <0.00001) (Figure 2). Subgroup analysis of OS by region revealed that the correlation between PNI and OS remained significant in China, Turkey, Japan, and the UK (Figure 3). Furthermore, significant heterogeneity decreased to 0% in the Turkish and Japanese subgroups (Figure 3). On the other hand, subgroup analysis of OS based on the PNI cutoff value revealed that the correlation between PNI and OS remained significant in both the cutoff value > 45 and the cutoff value ≤ 45 subgroups (Figure 4). Significant heterogeneity decreased to 22% in the cutoff value ≤ 45 subgroup (Figure 4). Because the studies by Fang et al. (30) and Yamaguchi et al. (45) did not report a specific PNI cutoff value, the subgroup comparisons in Figure 4 were not included in this analysis.

### Prognostic value of PNI for PFS

**Figure 5:**
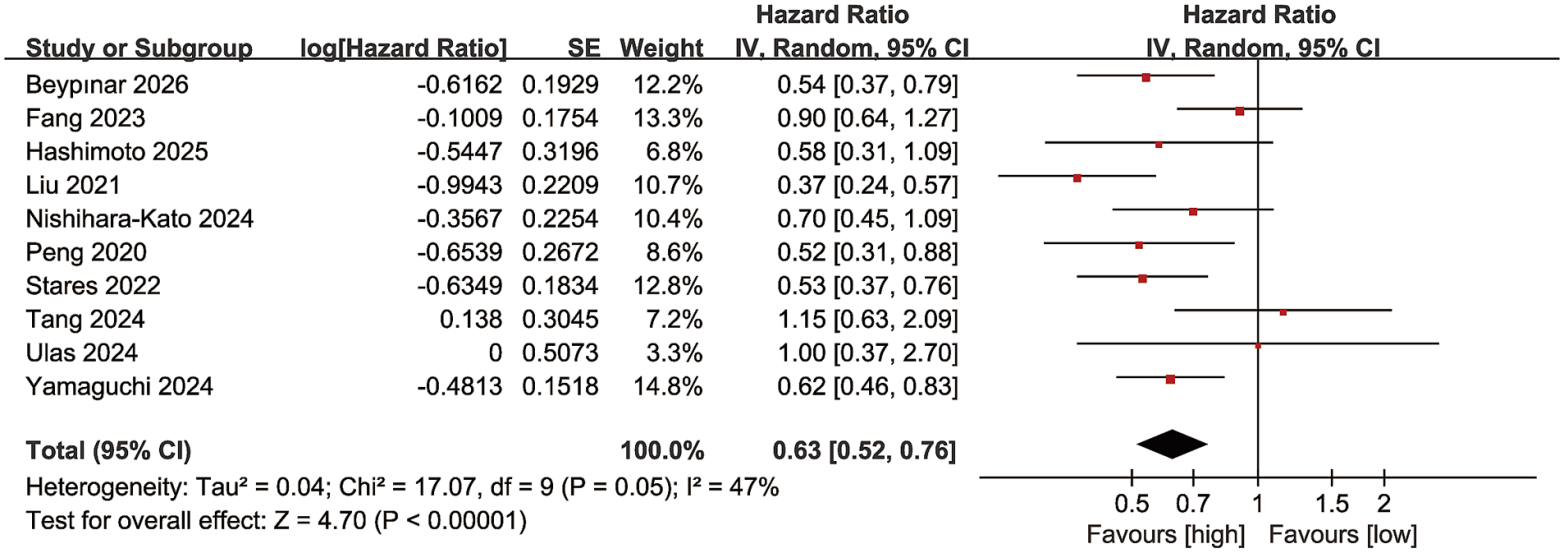
Forest plots of the prognostic value of PNI for PFS.

PFS results were synthesized from 10 studies. The meta-analysis indicated that a higher PNI was significantly associated with longer PFS (HR: 0.63; 95% CI: 0.52, 0.76; *p* <0.00001), without substantial heterogeneity (*I*^2^ = 47%, *p* = 0.05) (Figure 5).

### Publication bias

**Figure 6:**
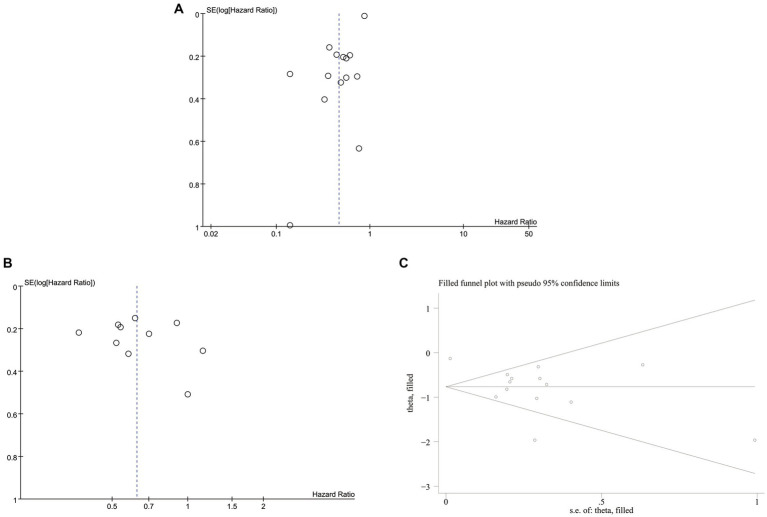
**(A)** Funnel plot of OS, **(B)** Funnel plot of PFS, and **(C)** Trimming and filling funnel plot of OS.

Potential publication bias regarding the prognostic significance of PNI for OS and PFS was evaluated using Egger’s regression test and funnel plot analysis. The results indicated the presence of significant publication bias for OS (Egger’s test *p* = 0.0001, Figure 6A). Egger’s test and funnel plot did not detect publication bias in PFS (Egger’s test *p* = 0.626, Figure 6B). The impact of publication bias on OS was assessed using the trim-and-fill method. The results indicated that the significant correlation between PNI and OS did not change after trimming and filling, suggesting that OS was not affected by publication bias (HR: 0.47; 95% CI: 0.34, 0.64) (Figure 6C).

### Sensitivity analysis

**Figure 7:**
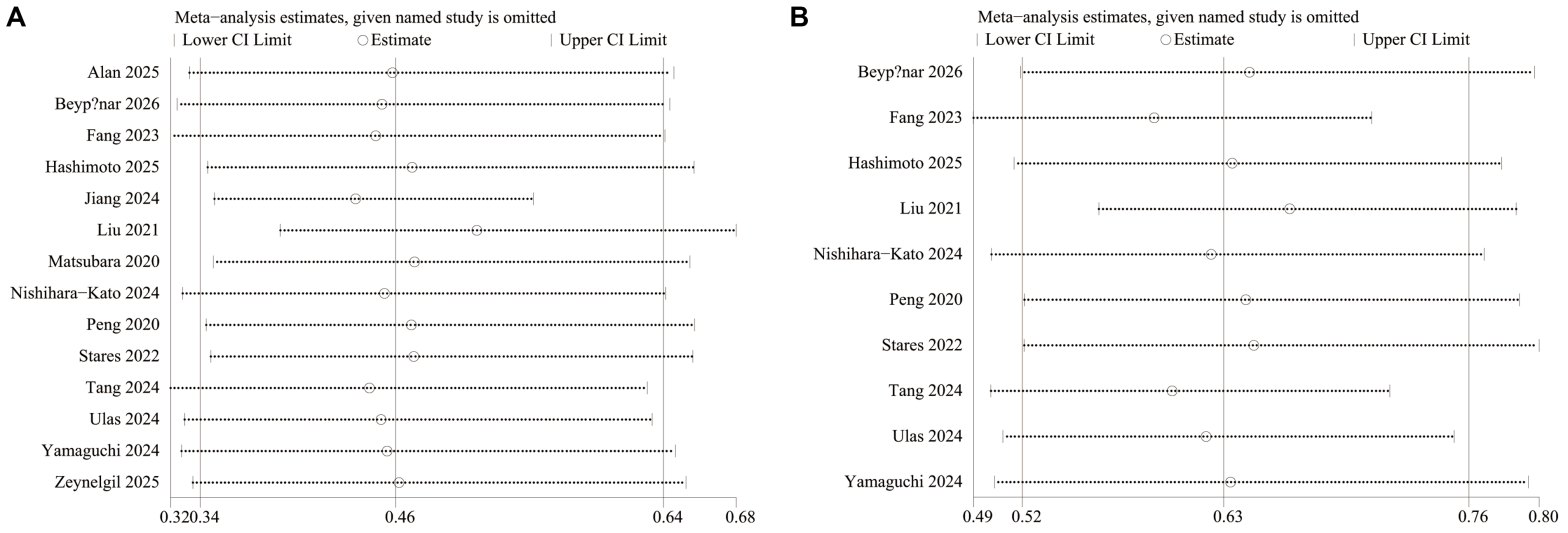
Sensitivity analysis of the prognostic value of PNI for **(A)** OS, **(B)** PFS.

A sensitivity analysis was performed to assess each study’s effect on the overall HR for the prognostic value of PNI for OS, and PFS using a leave-one-out approach. The overall HRs for OS (Figure 7A) and PFS (Figure 7B) remained consistent following the sequential exclusion of individual studies.

## Discussion

The main results of this meta-analysis indicate that a higher PNI is associated with significantly prolonged OS and PFS, providing evidence for PNI as a prognostic biomarker for effective immunotherapy in NSCLC. PNI is calculated based on serum albumin and peripheral blood lymphocyte count, both of which are readily available and inexpensive blood tests with a potential pathophysiological basis for their prognostic value. Serum albumin levels are a comprehensive indicator reflecting the body’s nutritional status, synthetic function, and negative regulatory capacity for systemic inflammation (46, 47). Hypoalbuminemia is often associated with cancer cachexia, chronic inflammation, and poor performance status, which directly or indirectly impair patients’ treatment tolerance and overall survival (48). More importantly, in the context of immunotherapy, lymphocytes, especially CD8^+^ cytotoxic T cells, are key effector cells for the anti-tumor effects of PD-1/PD-L1 inhibitors. A higher baseline lymphocyte count may indicate that patients have a stronger “immune reserve” and a more active potential for tumor immune infiltration, thus leading to a better response to immunosuppressive therapies (44, 45). Therefore, a high PNI value suggests that the patient may be in a favorable state with relatively good nutritional status, controlled systemic inflammation, and sufficient effector immune cells (43). This state is likely to create an internal environment in which immune checkpoint inhibitors are more effective, thus achieving more durable disease control and survival benefits. Conversely, a low PNI, representing malnutrition-inflammation-lymphopenia syndrome, may indicate an impaired or exhausted immune response, leading to primary or secondary resistance to immunotherapy (49).

Although PNI showed good overall predictive value for OS and PFS, we observed significant methodological heterogeneity in our analysis, particularly regarding OS. This high heterogeneity suggests substantial differences in effect sizes among different studies, the sources of which require careful analysis. Heterogeneity may stem from several factors: First, differences in study populations and treatment regimens. Included studies covered patients receiving different PD-1/PD-L1 inhibitors and different treatment regimens. Second, differences in PNI cutoff values. The PNI cutoff values used in the studies ranged from 40 to 46.67. This inconsistency in the definition of “high” and “low” PNI is a significant reason for the heterogeneity that makes direct comparison and pooling of effect estimates difficult. Therefore, we conducted subgroup analyses based on cutoff values. Interestingly, the analysis showed that the significant association between PNI and OS was maintained regardless of whether a cutoff value >45 or ≤45 was used, but heterogeneity decreased significantly to 22% in the subgroup with a cutoff value ≤45. This suggests that under a relatively stricter definition of “low PNI,” the prognostic role of PNI may be more consistent across different studies. Another important source of heterogeneity is geographical or population differences. Our subgroup analysis showed that the prognostic value of PNI was significant in the Chinese, Turkish, Japanese, and British populations, but there was no heterogeneity among studies in the Turkish and Japanese subgroups. This may reflect higher population homogeneity within these regions or relatively standardized medical practices. The Chinese subgroup still exhibited higher heterogeneity, possibly related to the large sample size, numerous centers, and high patient heterogeneity in Chinese studies.

In assessing the robustness of the evidence and potential bias, we conducted a series of supplementary analyses. Sensitivity analysis showed that excluding any individual study, whether OS or PFS, did not cause a directional change or loss of significance in the pooled HR, confirming the robustness of our primary conclusions and their independence from the undue influence of any single study. However, publication bias assessment suggested a significant publication bias in the OS analysis. To assess the impact of this bias on our conclusions, we used a pruning and imputation method for correction. Encouragingly, even after simulating the imputation of potentially missing negative studies, the significant negative correlation between PNI and OS remained significant, with confidence intervals overlapping with the uncorrected values. This result strengthens the credibility of our findings, indicating that despite publication bias, the core conclusion that PNI is a predictor of OS is reliable and unlikely to be overturned by potential unpublished data.

The findings of this study are consistent with previous meta-analyses (31), confirming that PNI is a potential predictor of survival in lung cancer patients receiving immunotherapy. However, our study significantly expands and deepens the analysis in terms of analytical dimensions, methodological depth, and clinical guidance. Compared to previous studies, our analysis has the following key features: First, we strictly limited the population to NSCLC patients, avoiding potential dilution or confounding effects of pathological heterogeneity on the predictive efficacy of PNI. Second, at the subgroup analysis level, our study further explored two novel and important stratification factors: study location and PNI threshold. Third, methodologically, we corrected for significant publication bias using a trimming-and-filling method, confirming the stability of the core associations. This is an important supplement to the analytical methods of previous studies, enhancing the reliability of the evidence. Finally, our study included the latest literature up to 2026, ensuring the timeliness of the evidence. Therefore, our meta-analysis not only validates and consolidates the cornerstone status of PNI in predicting the prognosis of lung cancer immunotherapy, but also strengthens the methodological rigor by focusing on a specific population, analyzing new sources of heterogeneity, and enhancing methodological rigor. Our findings provide updated, more targeted, and higher-level evidence for PNI as a practical tool for the personalized management of NSCLC immunotherapy.

Our results corroborate the prognostic value of other systemic inflammatory markers, such as the systemic immune inflammatory index (SII) (14, 50) and neutrophil-lymphocyte ratio (NLR) (51), in NSCLC immunotherapy, jointly emphasizing the importance of assessing patients’ systemic inflammatory and immune status before treatment. The unique advantage of PNI lies in its inclusion of both nutritional and immune dimensions, potentially providing more comprehensive information than indicators that simply reflect inflammatory cell homeostasis (37, 40). However, it must be noted that the vast majority of studies included in this meta-analysis were retrospective. While this is the primary form of current evidence in this field, its inherent limitations, such as selection bias, information bias, and uncontrollable residual confounding, mean that our analysis primarily established a strong association between PNI and prognosis, rather than a causal relationship. Furthermore, most studies were from Asia, demonstrating the value of PNI in these populations, but the universality of its conclusions in other ethnic groups worldwide requires further validation.

It is important to note that the PNI should be understood more accurately as a composite marker reflecting systemic inflammation, physiological reserves, and immune homeostasis, rather than a comprehensive nutritional assessment tool. Its calculation is based solely on serum albumin and lymphocyte count, both of which are influenced by multiple factors including nutritional status, inflammation levels, hepatic synthetic function, and immune status. A low PNI often fails to distinguish whether its primary driver is inadequate intake, high metabolic expenditure, chronic disease activity, or simple immunosuppression. Therefore, the core clinical value of the PNI lies in its accessibility, rather than replacing specialized nutritional screenings targeting body composition, dietary intake, or nutrient absorption. When interpreting its prognostic significance, it should not be simply attributed to “malnutrition,” but rather its underlying complex pathophysiological network should be recognized.

This study also has some limitations. First, due to limitations in the original data reporting, we were unable to conduct more in-depth subgroup analyses or meta-regressions on some important potential effect modifiers. For example, stratification by PD-L1 expression level, presence of liver metastases, patient performance status, or whether combination therapy was received. These analyses are crucial for identifying which specific subgroups of patients have the most predictive value for PNI. Second, PNI is a static baseline indicator. We were unable to analyze whether dynamic changes in PNI during treatment have stronger predictive value than a single baseline measurement. Some studies have shown that an increase in PNI in the early stages of treatment may be associated with a better treatment response. Third, we primarily focused on survival outcomes. The existing data are insufficient for a reliable meta-analysis of the relationship between PNI and the objective response rate of immunotherapy or the incidence of immune-related adverse events, which are also important considerations in clinical decision-making.

Based on the above discussion, we propose the following outlook for future research directions and clinical practice. At the research level, there is an urgent need to conduct large-scale, multicenter, prospective observational studies or secondary analyses of existing clinical trial data. This will help prospectively validate the predictive value of PNI and determine the optimal cutoff value in different clinical scenarios. Research should focus on combining PNI with existing predictive models and evaluating its net clinical benefit through decision curve analysis, etc. Furthermore, exploring the predictive value of dynamic monitoring of PNI is also a promising direction. At the clinical practice level, the evidence from this study supports considering PNI as a simple and practical risk assessment tool for NSCLC patients before initiating immunotherapy. Clinicians can consider incorporating baseline PNI into a comprehensive assessment system when making treatment decisions. For patients with low PNI, it should be recognized that they may face a poorer survival prognosis. This suggests the need for closer follow-up monitoring and more aggressive supportive care. Simultaneously, when conditions permit, clinical studies exploring its inclusion in intensive treatment strategies should be considered. However, before using PNI as the sole basis for guiding treatment escalation or downgrading decisions, evidence from prospective interventional studies is still needed. Furthermore, the low cost and availability of PNIs make them particularly important for applications in resource-constrained healthcare settings.

## Conclusion

This systematic review and meta-analysis suggests an association between higher baseline PNI and improved survival outcomes in NSCLC patients treated with PD-1/PD-L1 inhibitors. Given that the evidence included in this study was all from a retrospective design and was heterogeneous, these findings should be considered hypothesis-generating. As a low-cost, comprehensive indicator, the PNI must be validated in prospective studies for its incremental predictive value independent of known prognostic factors before being incorporated into routine clinical decision-making, and its clinical utility must be assessed using tools such as decision curve analysis.

## Data Availability

The original contributions presented in the study are included in the article/Supplementary material, further inquiries can be directed to the corresponding author.
