# Supplementary material for: Association between prognostic nutritional index and prognosis in non-small cell lung cancer patients receiving PD-1/PD-L1 inhibitors: a meta-analysis
Source: Front Nutr. 2026 Jul 23;13:1859449. doi: 10.3389/fnut.2026.1859449 (PMC13441826; doi:10.3389/fnut.2026.1859449)
Supplement: Supplementary file 1 [file Data_sheet_2.pdf]

PRISMA 2020 Checklist

Section/topic	Item No	Checklist item	Reported on Page Number/Line Number	Reported on Section/Paragraph
**TITLE**				
Title	1	Identify the report as a systematic review.	Page 1, Line 1	Title
**ABSTRACT**				
Abstract	2	See the PRISMA 2020 for Abstracts checklist (Table 2).	Page 2, Line 22	Abstract
**INTRODUCTION**				
Rationale	3	Describe the rationale for the review in the context of existing knowledge.	Page 4, Line 95	Introduction, second
Objectives	4	Provide an explicit statement of the objective(s) or question(s) the review addresses.	Page 4, Line 113	Introduction, third
**METHODS**				
Eligibility criteria	5	Specify the inclusion and exclusion criteria for the review and how studies were grouped for the syntheses.	Page 5, Line 132	Methods, second
Information sources	6	Specify all databases, registers, websites, organisations, reference lists and other sources searched or consulted to identify studies. Specify the date when each source was last searched or consulted.	Page 5, Line 123	Methods, first paragraph
Search strategy	7	Present the full search strategies for all databases, registers and websites, including any filters and limits used.	Page 5, Line 123	Methods, first paragraph
Selection process	8	Specify the methods used to decide whether a study met the inclusion criteria of the review, including how many reviewers screened each record and each report retrieved, whether they worked independently, and if applicable, details of automation tools used in the process.	Page 5, Line 123	Methods, first paragraph
Data collection process	9	Specify the methods used to collect data from reports, including how many reviewers collected data from each report, whether they worked independently, any processes for obtaining or confirming data from study investigators, and if applicable, details of automation tools used in the process.	Page 6, Line 147	Methods, third paragraph
Data items	10a	List and define all outcomes for which data were sought. Specify whether all results that were compatible with each outcome domain in each study were sought (e.g. for all measures, time points, analyses), and if not, the methods used to decide which results to collect.	Page 6, Line 147	Methods, third paragraph
	10b	List and define all other variables for which data were sought (e.g. participant and intervention characteristics, funding sources). Describe any assumptions made about any missing or unclear information.	Page 6, Line 147	Methods, third paragraph

Study risk of bias assessment	11	Specify the methods used to assess risk of bias in the included studies, including details of the tool(s) used, how many reviewers assessed each study and whether they worked independently, and if applicable, details of automation tools used in the process.	Page 6, Line 154	Methods, fourth paragraph
Effect measures	12	Specify for each outcome the effect measure(s) (e.g. risk ratio, mean difference) used in the synthesis or presentation of results.	Page 6, Line 161	Methods, fifth paragraph
Synthesis methods	13a	Describe the processes used to decide which studies were eligible for each synthesis.	Page 6, Line 161	Methods, fifth paragraph
	13b	Describe any methods required to prepare the data for presentation or synthesis, such as handling of missing summary statistics, or data conversions.	Page 6, Line 161	Methods, fifth paragraph
	13c	Describe any methods used to tabulate or visually display results of individual studies and syntheses.	Page 6, Line 161	Methods, fifth paragraph
	13d	Describe any methods used to synthesize results and provide a rationale for the choice(s). If meta-analysis was performed, describe the model(s), method(s) to identify the presence and extent of statistical heterogeneity, and software package(s) used.	Page 6, Line 161	Methods, fifth paragraph
	13e	Describe any methods used to explore possible causes of heterogeneity among study results.	Page 6, Line 161	Methods, fifth paragraph
	13f	Describe any sensitivity analyses conducted to assess robustness of the synthesized results.	Page 6, Line 161	Methods, fifth paragraph
Reporting bias assessment	14	Describe any methods used to assess risk of bias due to missing results in a synthesis (arising from reporting biases).	N/A	N/A
Certainty assessment	15	Describe any methods used to assess certainty (or confidence) in the body of evidence for an outcome.	N/A	N/A
**RESULTS**				
Study selection	16a	Describe the results of the search and selection process, from the number of records identified in the search to the number of studies included in the review, ideally using a flow diagram.	Page 7, Line 175	Results, first paragraph
	16b	Cite studies that met many but not all inclusion criteria ('near-misses') and explain why they were excluded.	Page 7, Line 175	Results, first paragraph
Study characteristics	17	Cite each included study and present its characteristics.	Page 7, Line 175	Results, first paragraph
Risk of bias in studies	18	Present assessments of risk of bias for each included study.	Page 7, Line 175	Results, first paragraph
Results of individual studies	19	For all outcomes, present, for each study: (a) summary statistics for each group (where appropriate) and (b) an effect estimate and its precision (e.g. confidence/credible interval), ideally using structured tables or plots.	Page 7, Line 175	Results, first paragraph

Results of syntheses	20a	For each synthesis, briefly summarise the characteristics and risk of bias among contributing studies.	Page 7, Line 184	Results, second paragraph
	20b	Present results of all statistical syntheses conducted. If meta-analysis was done, present for each the summary estimate and its precision (e.g. confidence/credible interval) and measures of statistical heterogeneity. If comparing groups, describe the direction of the effect.	Page 7, Line 184	Results, second paragraph
	20c	Present results of all investigations of possible causes of heterogeneity among study results.	Page 7, Line 184	Results, second paragraph
	20d	Present results of all sensitivity analyses conducted to assess the robustness of the synthesized results.	Page 8, Line 204	Results, eighth paragraph
Reporting biases	21	Present assessments of risk of bias due to missing results (arising from reporting biases) for each synthesis assessed.	N/A	N/A
Certainty of evidence	22	Present assessments of certainty (or confidence) in the body of evidence for each outcome assessed.	N/A	N/A
**DISCUSSION**				
Discussion	23a	Provide a general interpretation of the results in the context of other evidence.	Page 8, Line 217	Discussion, first paragraph
	23b	Discuss any limitations of the evidence included in the review.	Page 13, Line 347	Discussion, seventh
	23c	Discuss any limitations of the review processes used.	Page 13, Line 347	Discussion, seventh
	23d	Discuss implications of the results for practice, policy, and future research.	Page 13, Line 366	Discussion, seventh
**OTHER INFORMATION**				
Registration and protocol	24a	Provide registration information for the review, including register name and registration number, or state that the review was not registered.	Page 5, Line 124	Methods, fifth paragraph
	24b	Indicate where the review protocol can be accessed, or state that a protocol was not prepared.	Page 5, Line 124	Methods, fifth paragraph
	24c	Describe and explain any amendments to information provided at registration or in the protocol.	Page 5, Line 124	Methods, fifth paragraph
Support	25	Describe sources of financial or non-financial support for the review, and the role of the funders or sponsors in the review.	Page 14, Line 394	Funding, first paragraph
Competing interests	26	Declare any competing interests of review authors.	Page 14, Line 395	Conflicts of Interest, first paragraph
Availability of data, code and other materials	27	Report which of the following are publicly available and where they can be found: template data collection forms; data extracted from included studies; data used for all analyses; analytic code; any other materials used in the review.	N/A	N/A

Table 2 PRISMA 2020 for Abstracts checklist

Section/topic	Item No	Checklist item	Reported on Page Number/Line Number	Reported on Section/Paragraph
**TITLE**				
Title	1	Identify the report as a systematic review.	Page 1, Line 1	Title
**BACKGROUND**				
Objectives	2	Provide an explicit statement of the main objective(s) or question(s) the review addresses.	Page 2, Line 23	Abstract, first paragraph
**METHODS**				
Eligibility criteria	3	Specify the inclusion and exclusion criteria for the review.	Page 2 Line 27	Abstract, second
Information sources	4	Specify the information sources (e.g. databases, registers) used to identify studies and the date when each was last searched.	Page 2 Line 27	Abstract, second paragraph
Risk of bias	5	Specify the methods used to assess risk of bias in the included studies.	Page 2, Line 34	Abstract, second
Synthesis of results	6	Specify the methods used to present and synthesize results.	Page 2, Line 32	Abstract, second paragraph
**RESULTS**				
Included studies	7	Give the total number of included studies and participants and summarise relevant characteristics of studies.	Page 2, Line 36	Abstract, third paragraph
Synthesis of results	8	Present results for main outcomes, preferably indicating the number of included studies and participants for each. If meta-analysis was done, report the summary estimate and confidence/credible interval. If comparing groups, indicate the direction of the effect (i.e. which group is favoured).	Page 2, Line 36	Abstract, third paragraph
**DISCUSSION**				
Limitations of evidence	9	Provide a brief summary of the limitations of the evidence included in the review (e.g. study risk of bias, inconsistency and imprecision).	Page 2, Line 46	Abstract, fourth paragraph
Interpretation	10	Provide a general interpretation of the results and important implications.	Page 2, Line 48	Abstract, fourth paragraph
**OTHER**				
Funding	11	Specify the primary source of funding for the review.	N/A	N/A
Registration	12	Provide the register name and registration number.	N/A	N/A
