# Supplementary material for: Association between prognostic nutritional index and prognosis in non-small cell lung cancer patients receiving PD-1/PD-L1 inhibitors: a meta-analysis
Source: Front Nutr. 2026 Jul 23;13:1859449. doi: 10.3389/fnut.2026.1859449 (PMC13441826; doi:10.3389/fnut.2026.1859449)
Supplement: Supplementary file 2 [file Data_sheet_1.docx]

Pubmed-48

((("Carcinoma, Non-Small-Cell Lung"[Mesh]) OR (((((((Non-Small-Cell Lung Carcinomas) OR (Non-Small Cell Lung Cancer)) OR (Non-Small-Cell Lung Carcinoma)) OR (Non Small Cell Lung Carcinoma)) OR (Nonsmall Cell Lung Cancer)) OR (Non-Small Cell Lung Carcinoma)) OR (NSCLC))) AND (("Immune Checkpoint Inhibitors"[Mesh]) OR (((((((((((Programmed Cell Death Protein 1 Inhibitor) OR (PD-1 Inhibitor)) OR (PD 1 Inhibitor)) OR (Programmed Death-Ligand 1 Inhibitors)) OR (Programmed Death Ligand 1 Inhibitors)) OR (PD-L1 Inhibitor)) OR (PD L1 Inhibitor)) OR (PD-1)) OR (PD-L1)) OR (PD 1)) OR (PD L1)))) AND ((prognostic nutritional index[Title/Abstract]) OR (PNI))

Embase-75

((Carcinoma, Non-Small-Cell Lung or (Non-Small-Cell Lung Carcinomas or Non-Small Cell Lung Cancer or Non-Small-Cell Lung Carcinoma or Non Small Cell Lung Carcinoma or Nonsmall Cell Lung Cancer or Non-Small Cell Lung Carcinoma or NSCLC)) and (Immune Checkpoint Inhibitors or (Programmed Cell Death Protein 1 Inhibitor or PD-1 Inhibitor or PD 1 Inhibitor or Programmed Death-Ligand 1 Inhibitors or Programmed Death Ligand 1 Inhibitors or PD-L1 Inhibitor or PD L1 Inhibitor or PD-1 or PD-L1 or PD 1 or PD L1)) and (prognostic nutritional index or PNI)).af.

Cochrane-2

((Carcinoma, Non-Small-Cell Lung or (Non-Small-Cell Lung Carcinomas or Non-Small Cell Lung Cancer or Non-Small-Cell Lung Carcinoma or Non Small Cell Lung Carcinoma or Nonsmall Cell Lung Cancer or Non-Small Cell Lung Carcinoma or NSCLC)) and (Immune Checkpoint Inhibitors or (Programmed Cell Death Protein 1 Inhibitor or PD-1 Inhibitor or PD 1 Inhibitor or Programmed Death-Ligand 1 Inhibitors or Programmed Death Ligand 1 Inhibitors or PD-L1 Inhibitor or PD L1 Inhibitor or PD-1 or PD-L1 or PD 1 or PD L1)) and (prognostic nutritional index or PNI)).af.

Web of Science-73

(((Carcinoma, Non-Small-Cell Lung) OR (((((((Non-Small-Cell Lung Carcinomas) OR (Non-Small Cell Lung Cancer)) OR (Non-Small-Cell Lung Carcinoma)) OR (Non Small Cell Lung Carcinoma)) OR (Nonsmall Cell Lung Cancer)) OR (Non-Small Cell Lung Carcinoma)) OR (NSCLC))) AND ((Immune Checkpoint Inhibitors) OR (((((((((((Programmed Cell Death Protein 1 Inhibitor) OR (PD-1 Inhibitor)) OR (PD 1 Inhibitor)) OR (Programmed Death-Ligand 1 Inhibitors)) OR (Programmed Death Ligand 1 Inhibitors)) OR (PD-L1 Inhibitor)) OR (PD L1 Inhibitor)) OR (PD-1)) OR (PD-L1)) OR (PD 1)) OR (PD L1)))) AND ((prognostic nutritional index) OR (PNI)) (Topic)
